# Impact of Natural Compounds on Neurodegenerative Disorders: From Preclinical to Pharmacotherapeutics

**DOI:** 10.3390/jcm9041061

**Published:** 2020-04-08

**Authors:** Mehdi Sharifi-Rad, Chintha Lankatillake, Daniel A. Dias, Anca Oana Docea, Mohamad Fawzi Mahomoodally, Devina Lobine, Paul L. Chazot, Begum Kurt, Tugba Boyunegmez Tumer, Ana Catarina Moreira, Farukh Sharopov, Miquel Martorell, Natália Martins, William C. Cho, Daniela Calina, Javad Sharifi-Rad

**Affiliations:** 1 Department of Medical Parasitology, Faculty of Medicine, Kerman University of Medical Sciences, Kerman 7616913555, Iran; mehdi_sharifirad@yahoo.com; 2 School of Health and Biomedical Sciences, RMIT University, Bundoora, PO Box 71, VIC 3083, Australia; chintha.lankatillake@rmit.edu.au; 3 Department of Toxicology, University of Medicine and Pharmacy of Craiova, 200349 Craiova, Romania; daoana00@gmail.com; 4 Institute of Research and Development, Duy Tan University, Da Nang 550000, Vietnam; f.mahomoodally@uom.ac.mu or; 5 Department of Health Sciences, Faculty of Science, University of Mauritius, Réduit 80837, Mauritius; devinalobine@gmail.com; 6 Department of Biosciences, Durham University, Durham DH1 3LE, UK; paul.chazot@durham.ac.uk; 7 Graduate Program of Biomolecular Sciences, Institute of Natural and Applied Sciences, Canakkale Onsekiz Mart University, Canakkale 17020, Turkey; begum@stu.comu.edu.tr; 8 Department of Molecular Biology and Genetics, Faculty of Arts and Science, Canakkale Onsekiz Mart University, Canakkale 17020, Turkey; tumertb@gmail.com; 9 Pulmonology Department, Hospital Garcia de Orta, EPE Almada, 2801-951 Lisboa, Portugal; catarina_icbas@hotmail.com; 10 Department of Pharmaceutical Technology, Avicenna Tajik State Medical University, Rudaki 139, Dushanbe 734003, Tajikistan; shfarukh@mail.ru; 11 Department of Nutrition and Dietetics, Faculty of Pharmacy, University of Concepcion, Concepcion 4070386, Chile; martorellpons@gmail.com; 12 Universidad de Concepción, Unidad de Desarrollo Tecnológico, UDT, Concepcion 4070386, Chile; 13 Faculty of Medicine, University of Porto, Alameda Prof. Hernâni Monteiro, 4200-319 Porto, Portugal; 14 Institute for Research and Innovation in Health (i3S), University of Porto, 4200-135 Porto, Portugal; 15 Department of Clinical Oncology, Queen Elizabeth Hospital, Hong Kong, China; 16 Department of Clinical Pharmacy, University of Medicine and Pharmacy of Craiova, 200349 Craiova, Romania; 17 Phytochemistry Research Center, Shahid Beheshti University of Medical Sciences, Tehran 1991953381, Iran

**Keywords:** neurodegenerative disorders, Alzheimer’s disease, dementias, Parkinson’s disease, natural compounds, plants secondary metabolites, pharmacological activities

## Abstract

Among the major neurodegenerative disorders (NDDs), Alzheimer’s disease (AD) and Parkinson’s disease (PD), are a huge socioeconomic burden. Over many centuries, people have sought a cure for NDDs from the natural herbals. Many medicinal plants and their secondary metabolites are reported with the ability to alleviate the symptoms of NDDs. The major mechanisms identified, through which phytochemicals exert their neuroprotective effects and potential maintenance of neurological health in ageing, include antioxidant, anti-inflammatory, antithrombotic, antiapoptotic, acetylcholinesterase and monoamine oxidase inhibition and neurotrophic activities. This article reviews the mechanisms of action of some of the major herbal products with potential in the treatment of NDDs according to their molecular targets, as well as their regional sources (Asia, America and Africa). A number of studies demonstrated the beneficial properties of plant extracts or their bioactive compounds against NDDs. Herbal products may potentially offer new treatment options for patients with NDDs, which is a cheaper and culturally suitable alternative to conventional therapies for millions of people in the world with age-related NDDs.

## 1. Introduction

Neurological disorders (NDDs) are diseases that affect the central and peripheral nervous systems. NDDs can arise due to several factors, such as injury of the nervous system, ischaemia, oxidative and ER cellular stress, inflammation abnormal protein deposition in neural tissue, autoimmune-mediated neuronal loss and viral or prion infections [1]. Depending on the site affected, the neuronal loss, gliosis or demyelination can lead to motor deficits, behavioral disturbances and cognitive decline [2].

NDDs of the brain can be characterized by: memory loss or personality changes—Alzheimer’s disease (AD), impaired movement capacity and attention deficits—Parkinson’s disease (PD), weakness and cognitive decline—amyotrophic lateral sclerosis [1]. Peripheral nerve disorders include diabetic neuropathy, other metabolic neuropathies, endocrine neuropathies and disorders of myelin loss, with sensation deficits and autonomic dysfunction—Multiple sclerosis [1].

NDDs display severe impact to quality of life, characterized by a high disability-adjusted life years (DALY) (a measure of the loss of years of healthy life lost due to illness). Furthermore, the highest number of deaths due to NDDs is caused by stroke, placing it among the leading causes of non-traumatic death in industrialized countries [3,4,5]. The prevalence of NDDs is second only to headache disorders, and the global incidence of NDDs, such as AD and PD, are predicted to rise with population growth and increasing life expectancies [4]. Treatments currently available for NDDs are focused primarily on temporary symptomatic relief. Therefore, there is a high demand for the discovery of novel therapies and neuroprotective agents to prevent and retard the progression of NDDs [6]. Recently, transcranial magnetic stimulation (TMS) has been increasingly used as a non-invasive imaging technique for evaluating cortical function in patients with strokes and NDDs, to better understand the neurological changes produced and to apply a personalized treatment [7]. TMS has also showed efficacy in promoting clinical recovery after stroke and NDDs, the latter including vascular and post-stroke dementias, but this applicability is still in its infancy [1,8]. Other non-pharmacological approaches, for example, Shiatsu, physical activity, music therapy, have also showed beneficial effects for Quality of Life (QoL) in patients with several types of dementia [9,10]. The undesirable side-effects associated with some pharmacological compounds, used in conventional medicine, support the relevance of creating alternative therapies with higher efficacy and bioavailability, and fewer side-effects [11,12]. In this regard, plants can be a veritable source of novel compounds with therapeutic value for NDDs.

This article aims to review the beneficial role of plants and secondary metabolites in the prevention and management of NDDs. The first part of the discussion introduces secondary metabolites and focuses on their beneficial effects: antioxidants, anti-inflammatory, neuroprotective, antithrombotic, anti-acetylcholinesterase (AChE) and anti-monoamine oxidase (MAO) activities, while the second part focuses on the preclinical studies, and the effects of medicinal plants and their derived bioactive constituents on the pharmacotherapeutic management of NDDs

## 2. Methods

We conducted a PubMed search for the studies published between 2014 and 2019 using multiple combinations of keywords, including the following: natural compounds, flavonoids, bioflavonoid and neuroprotection, neurodegenerative diseases, Parkinson’s disease, Alzheimer’s disease. The review included only the relevant studies on the topic that used in vitro or in vivo models of AD and PD. Articles available only as abstract, bibliography, editorials, articles not written in English language and human studies were excluded.

## 3. Results and Discussion

**Figure 1:**
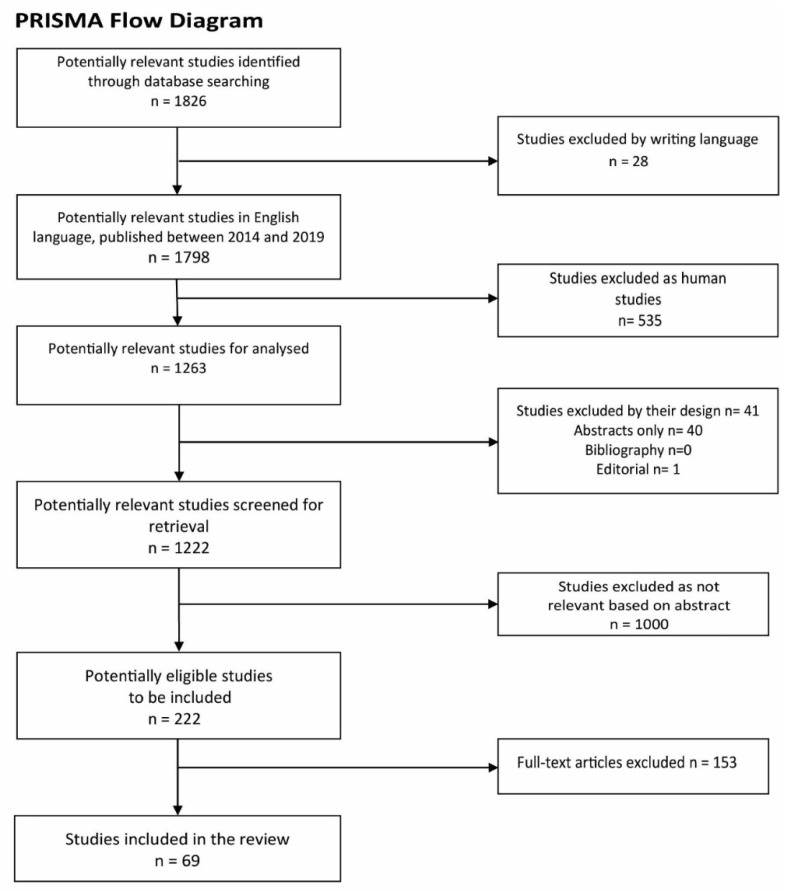
PRISMA flow diagram showing the search strategy, the number of records identified and the excluded and included articles [2].

The initial search identified 1826 publications on the topic, 28 studies were excluded at this stage, as they were not published in English. Checking the list, the human studies were excluded, that represented 535 studies, and, subsequently, 1263 potential eligible studies were considered. 41 studies based on their design and 1000 studies based on the relevance to the subject, respectively. Following abstract evaluation, 222 studies in full text, were analyzed. Of the 222 studies that were analyzed, 69 were finally included in the review. The selection procedure was performed according to the PRISMA (Preferred Reporting Items for Systematic Reviews and Meta-Analyses) flow chart [2] (Figure 1). 

### 3.1. Plants Secondary Metabolites: A Brief Overview

#### 3.1.1. Background

**Figure 2:**
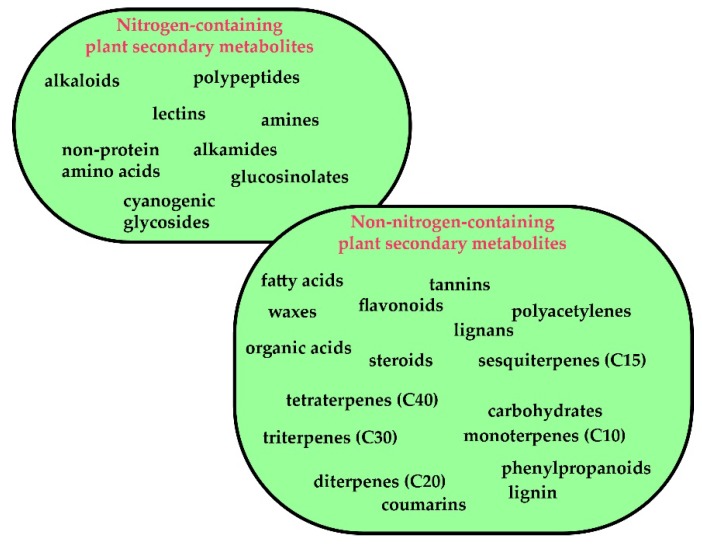
Types of secondary metabolites from plants.

Man’s reliance on nature for treating illness predates recorded history, and the plant kingdom is one of the most important sources of medicines [3,4,5,6,7,8,9]. The medicinal properties of plants are mostly ascribed to secondary phytochemical metabolites [10]. Secondary metabolites, also known as natural products, refer to small-molecule organic compounds that are not directly involved in growth and development, but play an adaptive role in aiding the survival of the organism [11,12,13,14,15]. Secondary metabolites are categorized into a number of compound classes, including terpenoids, alkaloids and phenylpropanoids and allied phenolic compounds, depending on their biosynthetic origins [11], or are broadly classified as nitrogen-containing or non-nitrogen-containing metabolites [10] (Figure 2).

Current evidence supports the applicability of natural compounds in human health, and their high diversity makes secondary metabolites a valuable source of novel drugs [16]. In fact, there is a growing interest in the potential use of secondary metabolites to prevent and manage NDDs. Some secondary metabolites may play protective roles in NDDs, including—but not limited to—neuroprotection from excitotoxicity, oxidative and ER stress, neuroinflammation and the prevention of proteinopathies [17,18].

#### 3.1.2. Ethnopharmacological Relevance of Natural Compounds for NDDs

Historical documents revealed that herbal therapies to treat NDDs date back to 6000 B.C. in India (Ayurvedic medicine), China, Africa and in Pre-Columbian America, especially with the Incas and the Aztecs [19]. Traditional herbal therapies still play an important role in the treatment of NDDs today. The Food and Drug Administration (FDA) consider herbal therapies as dietary supplements, rather than drugs. Complementary and alternative medicine (CAM) for NDDs have been widely used in most cultures. The National Institutes of Health National Center of Complementary and Alternative Medicine (NIH–NCCAM) defines CAM therapies as healthcare and medical practices that are not an integral part of conventional medicine as practiced in the west [20]. 

Herbal medicine is commonly used in Africa and up to 80% of the population uses traditional medicines as treatment [21]. In 2006, scientists predicted that the prevalence of NDDs in Sub-Saharan Africa was 2- to 3-fold higher than in developed countries [22]. In Africa, NDDs have a severe social impact, and patients face discrimination in education and employment [23]. Herbal drugs used for the treatment of NDDs included: *Alchornea laxiflora*, *Acanthusmontanus*, *Ficus platyphylla*, *Sutherlandia Frutescens*, *Gladiolus dalenii*, *Voacanga africana* [24]. 

In America, the pre-Columbian cultures, particularly Incas and Aztecs, have used more than 1500 plants to treat NDDs according to Spanish chroniclers [25]. The Aztec herbal textbook, “The Libellus de Medicinalibus Indorum Herbis” described the herbal treatments of NDDs and several plants were listed, such as *Bidenspilosa*, *Plucheaodorata*, *Lobelia laxiflora*, *Cassia occidentalis*, *Iresinecalea*, *Erythrina coralloides* and *Luffa operculate* [25,26]. In contemporary America, the herbal therapies are used in complementary or alternative medicine for many diseases and one in three patients with NDDs use herbal therapies. The most frequently used plant species for the treatment of NDDs in the United States are St. John’s wort (*Hypericum perforatum*), ginkgo (*Ginkgo biloba*), garlic (*Allium sativum*), black cohosh (*Actaearacemosa*), soy (*Glycine max*) and kava (*Piper methysticum*) [27]. 

In Asia, traditional Chinese medicine has a long history and recorded the use of herbal medicine in the treatment of NDDs. “The Yellow Emperor’s Classic of Internal Medicine” mentioned for the first time [28]. Chinese physicians believed that the balance between specific energies of the world; Yin and Yang determine the stability of the person’s health. If the balance is disturbed, NDDs will be manifested eventually [29]. For this reason, physicians worked to stabilize the balance between YIN and YANG by using herbs or acupuncture. The most preferred herbs are *Ginkgo biloba*, *Panax*, *Ganoderma lucidum*, *Salvia miltiorrhiza*, *Uncaria rhynchophylla* and *Zingibe rofficinale* [30]. 

The Indian medical system, Ayurveda (4500–1500 B.C.) “science of life”, is the oldest medical reference in the world [31]. In the Ayurvedic system, mechanisms within the human body were categorized by physiological and physicochemical activities and related to disease, including NDDs. Various herbal formulations are mentioned, including the amount of each component and the method of preparation [32]. The contents of these preparations include gandhaka (sulfur), butter oil and plants: *Ficus carica*, *Achythesaspena*, *Alstonia scholaris*, *Holanthena antidysenterica*. Some mixtures of herbal formulations, such as *Pancarnula* and *Triphala ahave* are included [33]. 

These ethnopharmacological uses of plants have guided scientific investigation for a large number of plant species, and has led to the identification of thousands of secondary metabolites, with desirable biological properties, including antioxidant, antimicrobial [34,35], anticancer [36,37], antidiabetic [38], anti-inflammatory [39] and neuroprotective properties [40]. 

### 3.2. Pharmacological Activities of Plants Secondary Metabolites on Neurodegenerative Disorders (NDDs): in vitro and in vivo Studies.

**Figure 3:**
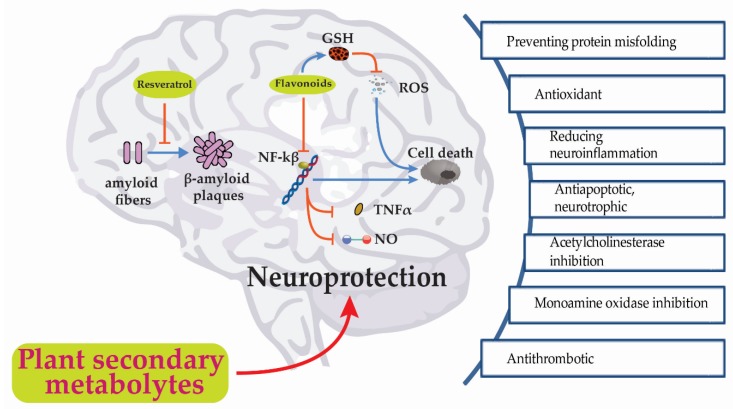
Summarized neuroprotective effects of plants secondary metabolites: Resveratrol may inhibit the formation of amyloid plaques from Alzheimer’s disease (AD); flavonoids stimulate the formation of glutathione (GSH), a powerful antioxidant that inhibits the formation of Reactive Oxygen Species (ROS) and participates in the defense of cells against oxidative damage. Flavonoids also inhibit Nuclear factor*-*κB *(*NF*-*κB), Tumor necrosis factor*-*α (TNFα), thus, preventing inflammatory-induced neuronal death. Symbols: ↑ stimulation; ↓ inhibition.

Recent studies have revealed that polyphenolic compounds, including flavonoids, phenolic acids and stilbenes; alkaloids, carotenoids, catechins and terpenes have great potential in treating NDDs (Table 1). Secondary metabolites with multiple beneficial effects on neurological health deserve special attention as they demonstrate the ability to act simultaneously on various targets and may assist in treating disorders with complex pathophysiologies (Figure 3).

#### 3.2.1. Preventing Protein Misfolding and Aggregation

Protein misfolding is a key pathological aspect of NDDs, such as AD, Huntington’s disease (HD) and PD [41] (Figure 3). In AD, the formation of extracellular senile plaques due to the accumulation of amyloid-*β* aggregates and neurofibrillary tangles (NFT) of tau proteins is associated with synaptic dysfunction, neuroinflammation and loss of neurons. Under normal physiological conditions, tau protein is involved in stabilizing microtubules. In AD, however, hyperphosphorylation of tau protein causes protein aggregation and the formation of intracellular NFT and the resultant degeneration of dendrites and axons [42]. Similarly, PD is characterized by the presence of the α-synuclein aggregates (Lewy bodies and Lewy neurites), the majority of which are found within the substantia nigra pars compacta (SNc) region of the midbrain. These α-synuclein aggregates acquire neurotoxic properties and compromise neuronal function and survival such as mitochondrial dysfunction, lysosome dysfunction, disruption of axonal transport and microglial activation leading to neuroinflammation [43]. Therefore, the prevention of proteinopathies is a strategy for treating NDDs. 

A number of secondary metabolites are credited with the ability to prevent aggregation of Aβ and reduce Aβ burden in experimental models of AD: the flavonoids apigenin [44], baicalein [45], hesperidin, isoquercetin, morin [46], narirutin [47] and quercetin [48]; the alkaloid berberine found in plants from the genus *Berberis* [49]; the carotenoid curcumin from *Curcuma longa* [50]; the catechin epigallocatechin gallate (EGCG) present in tea (*Camellia sinensis)* [51,52]; the stilbenoid resveratrol which is found primarily in grape skin and wine [53]; the monoterpene linalool, a major constituent in the essential oils (EOs) of a number of aromatic species such as lavender, rosemary and lemon balm [54]; withanolides from *Withania somnifera*, a medicinal plant also known as ‘Ashwadhanda’ in Ayurvedic medicine [44]. Curcumin also destabilizes Aβ aggregates and promotes disaggregation of existing Aβ deposits [50]. The reduction in Aβ aggregation is usually mediated via the inhibition of *β*-secretase (BACE1) and *γ*-secretase, enzymes involved in the processing of amyloid precursor protein (APP): berberine, quercetin, hesperidin [55] and narirutin [47], two secondary metabolites abundant in *Citrus* species, such as oranges and grapefruit, are inhibitors of BACE1; ginsenoside Rg1 from *Panax ginseng,* a widely used plant in Chinese medicine [49], isoquercetin and morin [46], act via the inhibition of both *β*-secretase (BACE1) and *γ*-secretase. Crocin, the principle carotenoid from saffron (*Croscus sativus*) inhibited tau hyperphosphorylation in the cerebral cortex of a rodent model of AD [56]. Isoquercetin, morin [46], linalool [54] also reduce tauopathy by inhibiting tau protein hyperphosphorylation. Quercetin was shown to block tau hyperphosphorylation by upregulating adenosine monophosphate-activated protein kinase (AMPK) and inhibiting glycogen synthase kinase 3 beta (GSK3*β*) [57]. The secondary metabolites with reported protective effects against synucleinopathy in PD include aegeline; an alkaloid-amide from *Aegle marmelos* [58], curcumin [50], EGCG, ginsenosides and withanolide A (from *W. somnifera*). The mechanism of inhibition of α-synuclein aggregation by these compounds remains unclear, and further investigation is necessary to reach conclusive results. 

#### 3.2.2. Antioxidant Activity

The brain is highly susceptible to oxidative stress (OS) due to its high metabolic activity, elevated oxygen requirement and the presence of high levels of redox-active metals and oxidizable lipids [59]. OS is an important mechanism involved in the pathogenesis and progression of many NDDs [60] (Figure 3). For example, mitochondrial injury and disrupted energy metabolism during cerebral ischemia and reperfusion generate nitric oxide (NO), and reactive oxygen species (ROS), subjecting the brain to an acute OS insult [61]. Aβ and cell damage induces the chronic production of ROS in the brains of AD patients. In PD, dopamine metabolism, mitochondrial dysfunction [62] and the neurotoxic effects of abnormal accumulation of α-synuclein, all promote the generation of ROS [63]. Furthermore, evidence suggests that there is reduced activity of endogenous antioxidant systems in PD patients [64]. Therefore, antioxidation is a major way by which phytochemicals exert their neuroprotective effects, which is a vital defense mechanism for neurological health. 

Antioxidant activity has been previously reported in a wide range of phytochemicals, with apigenin, baicalein, berberine, crocin, curcumin, ginsenosides, quercetin, resveratrol and rutin being some of the most effective [15,65,66,67]. Baicalein attenuated 6-hydroxydopamine (6-OHDA)-induced neurotoxicity (a common model of PD) by minimizing mitochondrial dysfunction and OS, and prevented NO production, by inhibiting inducible nitric oxide synthase (iNOS) in microglia [45]. Resveratrol also reduced NO and iNOS expression induced by Aβ in glial cells [53]. Berberine [49] and crocin decreased the expression of cyclooxygenase 2 (COX2) and iNOS in vitro and in vivo*,* respectively, and protected neurons from oxidative injury [68]. Quercetin promoted mitochondrial biogenesis, thereby protecting neurons from mitochondrial dysfunction-related ROS production and thus enhancing neuronal survival [40]. 

Acacetin [69], asiatic acid from *Cantella asiatica* [70], apigenin [44], ginsenosides, naringenin common in *Citrus* species [71] and rutin [72] reduced ROS generation. Rutin [72], asiatic acid [70] and ginsenosides [73] also attenuated mitochondrial dysfunction, and naringenin was observed to upregulate endogenous antioxidant enzymes [71]. The enhancement of endogenous antioxidant systems is often mediated via the activation of the nuclear factor-like 2 (Nrf2) signaling pathway which regulates the gene expression of antioxidant enzymes. Luteolin [54]; sulforaphane, an isothiocyanate found in cruciferous vegetables [15,74]; and naringenin [71] are examples of secondary metabolites with demonstrated Nrf2 activation properties in neuronal tissue. These secondary metabolites protect neurons from toxicity caused by a range of agents, including hydrogen peroxide, rotenone, Aβ and copper overload [40,44,70]. 

Epileptic seizures are common underappreciated symptoms found in many NDDs, including AD. The kindling model of epilepsy is a common model for studying epileptogenesis and the cerebral effects of multiple seizures. Kindling is generally induced by repeated focal stimulation of the brain [75]. The effects of curcumin supplementation on a model of neurological disorders in rats has been reported [76]. Cortical and hippocampal neurons were protected from seizure-induced death, this being attributable to the restoration of glutathione (GSH) levels in the brain. The authors also found that curcumin prevented seizure-induced mitochondrial dysfunction and damage of the mitochondrion ultrastructure in cortical and hippocampal neurons [76]. Luteolin increased GSH levels and lowered MDA (malondialdehyde, a marker of lipid peroxidation) in the pentylenetetrazole (PTZ)-induced seizure model of mice, demonstrating neuroprotection [77]. The restoration of GSH by curcumin and luteolin signifies an increase in endogenous antioxidant defenses, which in turn prevents protein oxidation and mitochondrial swelling, by attenuating oxidative injury to mitochondrial membranes [75] D. The activation of microglia is another prominent source of ROS and reactive nitrogen species (RNS). Baicalein [45], ginsenosides [78], linalool [54] and rutin [72] have demonstrated the ability to block the activation of microglia in in vitro and in vivo models of NDDs. Naringin, a flavanone-glycoside commonly found in *Citrus* plants, downregulated the expression of the glial fibrillary acidic protein (GFAP), which in turn reduced microglial activation in a rodent model of PD [48]. 

#### 3.2.3. Anti-inflammatory Activity

Neuroinflammation is a factor that plays an essential role in the increased loss of neuronal tissue and brain damage in cerebral ischemia, as well as in inflammatory diseases of the central nervous system (CNS) (Table 1). For example, neuronal damage due to the accumulation of Aβ, tau protein and α-synuclein results in the recruitment and activation of microglia, which initiates the inflammatory response [43,79,80]. Cerebral ischemia and reperfusion injury (CI/RI) trigger the recruitment of resident microglia as well as infiltrating macrophages and neutrophils, which is followed by an enhanced inflammatory response and an over-production of inflammatory mediators (cytokines) [81,82]. The inhibition or downregulated expression of key pro-inflammatory mediators and/or the upregulation of anti-inflammatory cytokines are beneficial for preventing chronic inflammation and further cell death [83].

The neuroprotective properties of secondary metabolites can be attributed, in part, to their antioxidant capacity and anti-inflammatory potential. Genistein, a secondary metabolite of plant origin, has the structure and function similar to the primary female sex hormone, 17-beta-estradiol [84]. Genistein is capable of attaching itself to the receptive proteins of the female sex hormones and having estrogen-specific hormonal effects, even being able to replace them, triggering either an estrogenic or a regulatory hormonal impact [85]. To date, studies have shown that the anti-inflammatory activity of curcumin, genistein, resveratrol and naringenin are arbitrated via the inhibition or downregulated translocation of nuclear factor-κB (NF-κB), which regulates the transcription of cytokines [80]. Ginsenosides [49], linalool [54], quercetin [57], resveratrol [53] and sulforaphane [74] inhibit pro-inflammatory cytokines such as tumor necrosis factor-alpha (TNF-α), interleukin (IL)-1β, IL-6 and IL-8 to reduce neuroinflammation in neurodegenerative models. In addition, quercetin also reduced GFAP in the brains of a rodent model of AD [57]. Curcumin [86] and kolaviron, a bioflavonoid complex isolated from *Garcinia kola* (bitter kola) [87], demonstrated neuroprotection from CI/RI by attenuating inflammation. Furthermore, secondary metabolites also attenuate neuroinflammation by preventing the activation of microglial cells [88].

#### 3.2.4. Antiapoptotic and Neurotrophic Activities

AD and PD are characterized by the primary loss of cholinergic and dopaminergic neurons in the neocortex and substantia nigra of the midbrain, respectively. The variety of clinical aspects of NDDs, such as cognitive, behavioral and movement deficits are the result of neurodegeneration and loss of neurons. Therefore, preventing neuronal cell death and promoting neuron survival and regeneration are key mechanisms for reducing the impact of NDDs. In addition to the function of protecting neurons against OS and inflammation, secondary metabolites are able to prevent apoptosis induced by various neurotoxic agents, prevent excitotoxicity and promote neurogenesis via the upregulation of neurotrophic factors [17,89] (Figure 3). Acacetin [69], baicalein [45] and ginsenosides [78] inhibited dopaminergic neuron loss and preserved locomotor activity and coordination in rodent models of PD. Baicalein [45], quercetin [57] and resveratrol have been shown to attenuate 6-OHDA-induced neurotoxicity. Resveratrol also displayed neurotrophic properties in rat hippocampus by activating extracellular signal-regulated kinases (ERK)1-2/ cAMP-response element-binding protein (CREB) pathways which increase brain-derived neurotrophic factors (BDNF) and glial cell line-derived neurotrophic factor (GDNF) [40] which contribute to synaptic plasticity in key parts of the brain. Resveratrol has anti-inflammatory and antioxidant properties and can reduce the oxidation and formation of amyloid plaques in AD. Therefore, it has the potential also of reducing the pathology of PD or HD, through similar mechanisms [90,91]. Baicalein has also been shown to prevent lipopolysaccharide (LPS)-induced inflammatory-based neurotoxicity [45], while asiatic acid [70] reduced apoptosis, and resveratrol prevented neurotoxicity by activating the AMP-activated protein kinase/sirtuin 1 (AMPK-SIRT-1) autophagy pathway (crucial for the orderly degradation and recycling of cellular protein components compromised in NDD proteinopathies) in rotenone-induced models of PD [40]. The neuroprotective effect of asiatic acid was also partially mediated by activation of the extracellular-signal-regulated kinase (ERK) and phosphatidylinositol 3 kinase/protein kinase B/mammalian target of rapamycin/glycogen synthase kinase 3 beta (PI3K/Akt/mTOR/GSK-3β) pathways. 

In experimental models of AD, apigenin, genistein, ginsenoside Rg1, isoquercitrin, morin, quercetin and rutin all protected cells from Aβ-induced apoptosis. Apigenin [44] and rutin [92] were seen to modulate mitogen-activated protein kinases (MAPK) activation. Ginsenoside Rg1 [49], isoquercetin [46] and morin [46] reduced caspase-3, and caspase-9 expression which inhibits apoptosis. Apigenin [44], sulforaphane [93] and withanolide A [89] exhibited neurotrophic properties in AD models. For sulforaphane, the neurotrophic mechanism of action was shown to be via increased expression of p75NTR (p75 neurotrophin receptor) [93], while apigenin restored the compromised ERK/CREB/BDNF pathway, thus promoting neuroplasticity mechanisms and neurogenesis [44]. 

Baicalein and ginsenosides were shown to protect neurons from cerebral ischemia injury [45]. Baicalein protected rat hippocampi from glutamate-induced neurotoxicity by chelating intracellular Ca^2+^, which reduces presynaptic glutamate release [45]. Ginsenoside Rd showed beneficial effects for strokes through increased neuron survival following CI/RI, reduced infarct volume and protection of neurons against excitotoxicity by upregulating the glial glutamate transporter 1 (GLT-1), thus enhancing glutamate clearance by astrocytes [73]. Kolaviron also reduced necrotic cell death in rats subjected to bilateral common carotid artery occlusion-induced global ischemia/reperfusion injury. Prevention of excitotoxicity by reducing the release of excitatory neurotransmitters, and the preservation of Na/K/ATPase pump activity, which assists with electrolyte balance, has been proposed to be partially responsible for this neuroprotective effect [87].

#### 3.2.5. Acetylcholinesterase Inhibition Activity

Acetylcholinesterase (AChE) inhibitors are a widely used class of drugs for treating early-stage dementias [94]. Acetylcholine (ACh), a neurotransmitter important for memory processing, is severely depleted in AD due to the degeneration of cholinergic neurons in the basal forebrain and, in particular, the nucleus basalis of Meynert which contributes to the cognitive impairment and memory loss seen in AD [95,96] (Figure 3). AChE is responsible for the postsynaptic degradation of ACh and, therefore, selective AChE inhibitors treat dementia by prolonging ACh activity. 

Galantamine, an alkaloid extracted from *Galanthus* species, is a currently prescribed AChE inhibitor drug for AD and dementia [94,97]. Other promising secondary metabolites of note with AChE inhibitory activity are crocin, EGCG and naringin. In a recent study, crocin improved memory by inhibiting AChE and increasing ACh levels in the cortex and hippocampus in an AD-model mouse [68]. Naringin [92] and EGCG [18] exhibited AChE inhibitory activity and restored cognitive function and memory in rodent models of AD and dementia, respectively.

#### 3.2.6. Monoamine Oxidase Inhibitors (MAOs)

Monoamine oxidases (MAO) are mitochondrial enzymes involved in the oxidative deamination of amine neurotransmitters [98,99]. In humans, two MAO isoforms are described, MAO-A and MAO-B, which share a degree of overlap between their substrate specificities. For example, both enzymes can metabolize dopamine, which makes them an important therapeutic target for PD [99,100]. MAO-A inhibitors have applications in the treatment of depression, anxiety and mood disorders as the enzyme subtype is also involved in the selective deamination of serotonin [98,100]. 

Molecular simulation studies have discovered chrysin, myricetin and genistein, an isoflavonoid from soybean, to possess strong inhibitory capacities versus MAO-A [99] (Figure 3). In a molecular docking study, the MAO-A-inhibitory potential of baicalein and decursin; two compounds isolated from the roots of *Scutellaria baicalensis* was reported [99]. In recent research, secondary metabolites, including berberine, maackiain and 3-phenyl coumarins were identified as novel MAO-B inhibitors [101]. 

#### 3.2.7. Antithrombotic Activity

Acute cerebral ischemia is usually initiated by thrombotic or thromboembolic obstruction of a cerebral artery, which causes a sudden drop in blood flow to part of the brain. The resultant hypoxia and reduction in glucose supply to neurons causes depletion of adenosine triphosphate (ATP) and irreparable damage to surrounding neurons [102] (Figure 3). Reducing the risk of thrombosis using antithrombotic or antiplatelet agents is a preventative strategy for ischemic stroke [80,103]. In addition, preventing thrombosis of collateral arteries can potentially improve cerebral perfusion and protect the ischemic penumbra from further post-ischemic damage [104]. 

Studies on the antithrombotic properties of secondary metabolites appear to be limited. However, recent reviews have highlighted that carvacrol, α-cyperone and nootkatone; terpene compounds from the essential oils (Eos) of *Cyperus rotundus* [105], showed antiplatelet aggregation activity. On occasion, natural derivatives with a coumarin structure may have anticoagulant and antithrombotic activity, via a vitamin K antagonist mechanism, which has relevance to vascular dementias. Coumarin is well absorbed in the digestive tract, binds to plasma proteins and is metabolized by the liver [106]. The response settles slowly, is dose-dependent, within the range of interindividual variations and influenced by some associated pathologies (liver disease, thyroid disease) as well as some foods or medicines. Thus, the consumption of large quantities of green vegetables, rich in vitamin K, will mitigate the anticoagulant effects while the consumption of alcohol or the combination of non-steroidal anti-inflammatories or of anti-platelet agents may increase the risk of bleeding [107]. 

Andrographolide, a diterepene-lactone from *Andrographis paniculata*, is reported to delay thrombus formation by activating endothelial nitric oxide-nitric oxide eNOS-NO/cyclic GMP pathways, which result in the downregulation of the phospholipase/protein tyrosine kinase (PLC-γ2/PKC) and PI3K/Akt/p38 MAPK cascades [104]. The inhibition of PLC-γ2/PKC and PI3K/Akt/p38 MAPK cascades block platelet activation and aggregation by preventing cytoplasmic Ca^2+^ mobilization necessary for the production of thromboxane A2, and inhibiting phosphorylation of PLA2 (phospholipase A 2) [108]. Wogonin from *Scutellaria baicalensis*, and the terpene-derivative borneol (found in a number of species, such as lavender, rosemary and Artemisia) also demonstrated anticoagulation activity. The anticoagulation activity of wogonin appears to be via the suppression of the synthesis and activity of thrombin and factor-Xa, which in turn disrupts coagulation pathways and reduces activated partial thromboplastin time (APTT), and prothrombin time (PT) [108]. 

## 4. Future Perspectives

The strength of this comprehensive and up-to-date study is the analysis of evidence from preclinical studies from a large number of meta-analyses related to the impact of natural bioactive compounds in NDDs. All these analyses focused on highlighting the molecular and cellular mechanisms of action to open new beneficial therapeutic perspectives in the therapy of NDDs. It is difficult to attribute the pharmacological activity of a plant/or plant extract to a single compound or class of compounds as the observed beneficial properties are often the result of synergistic effects between multiple compounds on multiple targets. Many of these compounds, for example, apigenin, crocin, curcumin, EGCG, ginsenosides, hesperidin, linalool, quercetin, resveratrol, rosmarinic acid and withanolides, are either common across many plant species, present in species used in traditional medicine, and/or found in food sources, such as fruits, herbs and spices. These findings highlight the contribution of traditional medicine to modern treatments, as well as the health benefits of dietary phytochemicals. 

An important aspect which limits the therapeutic applications of these natural compounds for NDD treatment is related to their poor bioavailability. For example, curcumin is a polyphenol with proven effects in the pharmacotherapy of AD, but with very low absorption and bioavailability. To increase its bioavailability, the resistance to metabolic processes and the passage through the blood-brain-barrier, new pharmaceutical technologies are required, such as liposomal nano-encapsulation, polymeric micelles, nanoparticles (nanocurcumin), cyclodextrins, nano-suspensions and nano-emulsions. Furthermore, additional properties of secondary metabolites, such as their metabolism, ability to cross the blood-brain-barrier, the dosage required for beneficial effects in humans, without toxic effects and interactions with current medications, are also important factors to be taken into consideration which need longitudinal investigations. It can be concluded that the plant secondary metabolites offer an abundant source of structurally and functionally diverse molecules for new potential preventative and therapeutic use in NDDs.

## Figures and Tables

**Table 1:** Summarized beneficial effects of plant secondary metabolites in the pharmacotherapy of neurodegenerative disorders.

Compound/Type	Natural Source	Experimental Model	Effects/Mechanisms of action	Ref
Acacetin/flavanoid	*Chrysanthemi indici*, *Calamintha*, *Linaria* spp	In vitro model of PD	↓6-hydroxydopamine-induced cell death ↓caspase-3, ↓caspase-9, ↓PARP and cytochrome c ↑Bcl-2/Bax, ↓ROS, ↓phosphorylation of JNK, ↓p38, ↓ERK1/2 MAPK	[69]
Aegeline/alkaloid-amide	*Aegle marmelos*	In vitro yeast model of PD	Prevented α-synuclein-induced apoptosis, ↓ROS	[58]
Andrographolide/diterpene lactone	*Andrographis paniculata*	In vitro model of PD	↓PAF-induced platelet aggregation, ↓collagen-stimulated platelet activation, ↑TXA2, ↑phosphorylation of PKC, MAPK and AKT ↑eNOS, ↑NO, ↑eNOS-NO/cyclic GMP pathways, ↓PI3K/Akt/p38 MAPK ↓ PLC-γ2/PKC	[104]
Apigenin/flavanoid	common constituent in plants	In vitro induced neurogenesis In vivo mouse model of AD	↓inflammatory cytokines, ↓cortical hyperexcitation ↓A*β* burden, ↓oxidative stress, ↑ERK/CREB/BDNF pathway ↓*β*-amyloid neurotoxicity, ↑mitochondrion protection	[44,92] [109]
Asiatic acid/triterpene	*Centella asiatica*	In vitro model of PD In vivo mouse model of PD	↓apoptosis, ↓ROS ↑ERK, ↑PI3K/Akt/mTOR/GSK-3*β* pathways, ↓MAPK/P38, ↓JNK, ↓ERK, ↓dopamine depletion, ↑NTFs	[110] [70]
Baicalein/flavanoid	*Scutellaria baicalensis*	Molecular docking simulation In vivo model of PD	↓MAO-A, ↓A*β* ↓brain hypoxia, ↓H_2_O_2_, ↓iNOS, ↓NF-*κ*B, ↓NO, ↓TNF-*α,* ↓oxidative stress, ↓mitochondrial dysfunction, ↑JNK, ↓TNF-*α,* ↓IL-6, ↓NF-*κ*B, ↓MAPK, ↓dopaminergic neuron loss, ↓LDH, ↓NO, ↓glutamate	[111] [45]
Berberine/alkaloid	*Berberis* genus	In vitro model of AD In vivo rodent model of AD	↓AChE, ↓MAO-B, ↓BACE1, ↑I*κ*B-*α*, ↑Akt,↑p38 kinase ERK1/2 ↓NF-*κ*B, ↓TNF-*α*,↓IL-6 production, ↓MCP-1, ↓COX 2,↓iNOS ↓A*β* plaque, ↓ CTF-*α*, ↓CTF-*β* (which reflects *α*- and *β*-secretase processing of APP)	[112] [101] [49]
Borneol/terpene derivative	common constituent in plants	Ex vivo rat blood	↑PT, ↑TT, ↓thrombosis in veins	[108]
Carvacrol/monoterpenoid phenol	*Cyperus rotundus*	In vitro MAO A and MAO B	↓antiplatelet aggregation	[105]
Chrysin/flavanoid	*Hypericum afrum,* *Cytisus villosus*	Molecular docking simulation	↓MOA-A	[99]
Crocin/carotenoid	*Gardenia jasminoides* *Crocus sativus*	In vivo mouse model of AD In vivo rat model of AD	↓oxidative stress, ↑SOD, ↓MDA ↓AChE, ↑ACh activity ↓neuroinflammation, ↓TNF-*α*, ↓PGE_,_ ↓iNOS, ↓COX2 ↓Tau hyperphosphorylation	[68] [56]
Curcumin/carotenoid	*Curcuma longa*	In vivo Dania rerio (zebrafish) model of NDD In vivo mouse model of stroke in vivo mice model of PD	Neuroprotective,↓tonic-clonic seizures ↓oxidative stress ↑GSH in cortex and hippocampus ↓infarct volumes, ↑M2 polarization of microglia/macrophages, ↓A*β* aggregation, ↓NF-*κ*B, ↓*α*-synuclein oligomerization	[113] [76] [86] [50,114]
Decursin/pyranocoumarin	*Angelica gigas*, *Scutellaria baicalensis*	In vitro model of PD	↓MOA-A	[111]
Epigallocatechin gallate/catechin	*Camellia sinensis*	In vivo rat model of AD rat model of PD	↓A*β* fibrillogenesis, ↓oxidative stress, ↓AchE ↓α-synuclein aggregation	[18,51]
Genistein/flavanoid	*Glycine max*	Molecular docking simulation In vitro model of AD	↓MAO ↓inflammation, ↓NF-*κ*B ↓A*β* toxicity, ↑apoptosis	[99] [44]
Ginsenoside Rd/triterpene glycosides	*Panax ginseng*	In vivo rodent model of stroke	↓excitotoxicity, ↓Ca^2+^ influx, ↑ GLT-1, ↓ ROS	[73]
Ginsenoside Rg1/triterpene glycosides	*Panax notoginseng*	In vitro cell model of AD	↓*β*- and γ-secretases, ↓NO, ↓ROS, ↓lipid peroxidation, ↓IL-1,↓IL-8, ↓TNF-*α*, ↓A*β* plaque, ↓caspase-9, ↓caspase-3	[49]
Hesperidin/flavanoid	*Valeriana officinalis*	Molecular docking simulation In vivo rat model of AD	↓ BACE1 ↓oxidative stress, ↓A*β* fibril formation	[109] [46,55]
Isoquercitrin/flavonoid	Common in plants	In vivo rat model of AD	↓BACE1, ↓γ-secretase, ↓A*β* fibrillogenesis, ↓caspase-3, ↓caspase-9, ↓apoptosis, ↓amyloid plaque, ↓tau hyperphosphorylation	[46]
Kolaviron/bioflavanoid complex	*Garcinia kola*	In vivo rat model of stroke	↓MPO, ↓necrotic cell death, Preserved Na/K/ATPase activity	[87]
Linalool/monoterpene	*Lavandula* spp. *Rosmarinus officinalis Melissa officinalis Cymbopogon citratus*	In vivo mouse model of AD In vitro cell model of ND	Anti-inflammatory ↓p38, ↓MAPK, ↓Nos2, ↓COX2, ↓IL-1*β*↓ A*β* in the hippocampus ↓tauopathy, inhibition of T-type Ca^2+^ channels	[54] [115]
Luteolin/flavanoid	Common constituent in plants	In vivo mouse model of ND In vivo animal model of stroke	↑GSH, ↓oxidative stress, ↓MDA, ↑Nrf2, antioxidant/anti-inflammatory ↑Nrf-2 dependent transcription of HO-1 neuroprotective against cerebral I/R injury	[77] [116] [82]
Morin/flavanoid	Common constituent in plants	In vivo rat models of AD	↓BACE1, ↓γ-secretase, ↓A*β* fibrillogenesis ↓apoptosis, ↑caspase-3, ↑caspase-9 ↓amyloid plaque, ↓tau hyperphosphorylation	[46]
Myricetin/flavanoid	Common constituent in plants	Molecular docking simulation	↓MAO	[99]
Naringenin/flavanoid	*Citrus paradise* *Citrus sinensis*	In vitro models of AD	↓inflammatory cytokines, ↓NF-*κ*B signalling, ↑Nrf2/ARE signaling ↓NO	[71]
Naringin/flavanoid	*Citrus* spp.	In vivo rat model of AD In vivo rodent model of PD	↓AChE, ↓cognitive deficit, ↓GFAP, ↑neurotrophic factors	[92] [48]
Narirutin/flavanoid	*Citrus* spp.	In vitro	↓BACE1 ↓A*β* aggregation	[47]
Nootkatone/sesquiterpene	*Cyperus rotundus*	In vitro MAOA and MAOB	↓platelet aggregation	[105]
Quercetin/flavanoid	Tea, citrus	Molecular docking simulation In vivo mouse model of AD In vivo rodent model of PD	↓MAO, ↓PKC-ε ↓oxidative stress by ERK1/2 phosphorylation, p38MAPK dephosphorylation, ↓TNF-*α*, ↓IL-6, ↓GFAP, ↓MDA, ↑glutathione peroxidase, ↑AMPK activity ↓apoptosis, ↓GSK3*β,* ↓tau phosphorylation, ↓ROS, ↓A*β* aggregation ↓ BACE1, ↑NF-*κ*B, ↓ROS, improved 6-OHDA-induced tremors	[99] [116] [48] [40] [57]
Resveratrol/stilbenoid	*Vitis vinifera*	In vivo rat model of PD In vivo rodent model of PD	↓COX2, ↓TNF-*α*, ↓NF-*κ*B, ↓*β*-amyloid plaques ↓TNF-*α,* ↓IL-6, ↑BDNF, ↑IL-10, ↓TNF-*α,* ↓NF-*κ*B ↑ERK1-2/CREB, ↑BDNF, ↑GDNF, ↓NO, ↓iNOS, ↓A*β* in glial cells, ↑AMPL-SIRT-1	[53] [40]
Rutin/flavanoid	Abundant in *Citrus* fruits	In vitro In vitro In vivo rodent model of AD	↓pro-inflammatory cytokines, ↓ROS Protected neurons against oxidative injury ↑SOD, ↑CAT, ↑GPx, ↓iNOS ↑MAPK, ↑apoptosis, ↑JNK, ↑p38 MAPK ↓ IL-1, ↓IL-6, ↑BDNP expression	[72] [92] [117] [92]
Silibinin/flavanoid	*Silybinisus laborinum*	In vivo rat model of AD In vivo rat model of PD In vivo rat model of stroke	↓AChE, ↓ROS ↓A*β* aggregation, ↓hypoxic/ischemic injury Protected neurons from H_2_O_2_-mediated oxidative stress ↓LC3-II, ↓Beclin-1 levels	[118] [57] [119]
Sulforaphane/isothiocynate	Cruciferous vegetables	In vitro cell model of AD In vivo mouse model of AD	↓IL-1*β*, ↓A*β*_1-42_-stimulated THP-1 macrophages Dephosphorylated STAT-1, ↑Nrf2 ↑neurogenesis, ↓aluminium load, ↓A*β* deposition ↑p75NTR, ↓A*β* burden	[74] [120] [93]
Withanamides A and C/amido compounds	*Withania somnifera*	In vivo rat model of AD	↓A*β* fibril formation	[44]
Withanolide A/amido compound	*Withania somnifera*	In vivo rat model of AD	↑axonal/dendritic regeneration exhibited neurotrophic activity	[89]
Withanone	*Withania somnifera*	In vivo rat model of AD	Protect neurons and glial cells	[121]
Wogonin/flavanoid	*Suctellaria baicalensis*	In vivo rat model of stroke	↓synthesis of thrombin, ↓factor-Xa ↓APTT, ↓PT	[108]
*α*-cyperone	*Cyperus rotundus*	In vivo rodent model of stroke	↓platelet aggregation	[105]

**Legend:** ↓-reducing, ↑-increasing, Alzheimer diseases (AD), Parkinson disease (PD), poly ADP ribose polymerase (PARP), c-Jun N-terminal kinase (JNK), extracellular signal-regulated, kinase 1/2 (ERK1/2) mitogen-activated protein kinase (MAPK) (ERK1/2 MAPK), reactive oxygen species (ROS), Protein Kinase C (PKC), serine/threonine kinase (Akt), Inducible nitric oxide synthase (iNOS), endothelial nitric oxide synthase (eNOS), nitric oxide (NO), guanosine monophosphate (GMP), phosphoinositide 3-kinase (PI3K)/serine/threonine kinase (Akt)/p38 mitogen-activated protein kinase (MAPK) (PI3K/Akt/p38 MAPK), phospholipase C γ2-protein kinase C (PLC-γ2/PKC), extracellular-signal-regulated kinase (ERK)/cAMP-response element binding protein (CREB)/Brain-derived neurotrophic factor (BDNF), phosphoinositide 3-kinase (PI3K)/threonine kinase (Akt)mammalian target of rapamycin (mTOR)/Glycogen synthase kinase 3 beta (GSK3β), 1-methyl-4-phenyl-1,2,3,6-tetrahydropyridine (MPTP), Monoamine oxidase A, (MAO-A), Amyloid β protein (Aβ), lipopolysaccharide (LPS), nuclear factor (NF-κB), tumor necrosis factor-α (TNFα), 6-hydroxydopamine (6-OHDA), Lactate dehydrogenase (LDH), Acetylcholinesterase (AChE), Beta-secretase 1 (BACE1), inhibitor of kappa B (IκBα), Monocyte chemoattractant protein-1 (MCP-1), cyclooxygenase-2 (COX-2), alpha-secretase (CTF-alpha) alpha-secretase (CTF-beta), Prostaglandin E2 (PGE2), pentylenetetrazol (PTZ), terleukin-1 beta *(*IL-1 *β),* interleukin (IL-8), tumor necrosis factor alpha (TNF-α*),* sulfonylurea receptor-1 (SUR1), myeloperoxidase (MPO), Malondialdehyde (MDA), erythroid 2-related factor (Nrf2), nuclear heme oxygenase-1 (HO-1), extracellular signal-regulated kinase ½ (ERK1/2), p38 mitogen-activated protein kinases (MAPKs), Protein kinase C epsilon typ PKC-ε, AMP-activated protein kinase (AMPK), glial fibrillary acidic protein (GFAP), glycogen synthase kinase 3β (GSK3β), brain-derived neurotrophic factor (BDNF), AMP-activated protein kinase/sirtuin 1 (AMPK/SIRT1), superoxide dismutase (SOD), catalase (CAT), glutathione peroxidase (GPX), p75 neurotrophin receptor (p75NTR), mitogen-activated protein kinase (MAPK), Jun N-terminal kinase (JNK), p38 mitogen-activated protein kinase (p38 MAPK), brain-derived neurotrophic factor (BDNF), microtubule-associated protein 1 light chain 3 (LC3), interleukin-1β (IL-1*β)*, the 42 amino acid form of amyloid β (Aβ1–42), signal transducer and activator of transcription 1 (STAT1), partial thromboplastin time (PTT), prothrombin time (PT), Thromboxane A2 (TXA2), glial glutamate transporter 1 (GLT-1).
